# Supplementary figures and images for: Idiopathic inflammatory myopathy human derived cells retain their ability to increase mitochondrial function
Source: PLoS One. 2020 Nov 20;15(11):e0242443. doi: 10.1371/journal.pone.0242443 (PMC7679003; doi:10.1371/journal.pone.0242443)

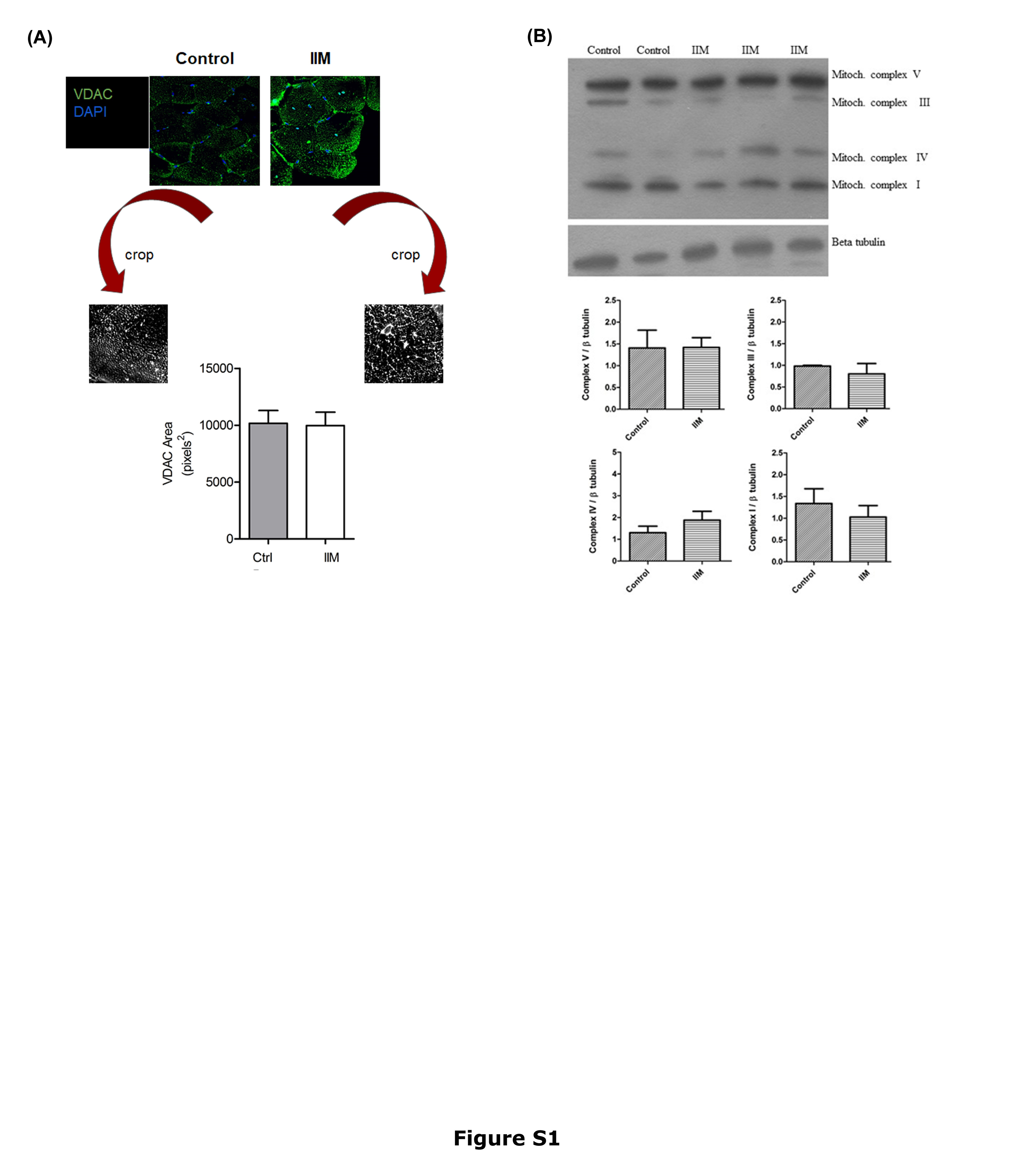

Supplement: S1 Fig — (A) Human skeletal muscle biopsies obtained from controls and IIM patients were labeled with a specific antibody against the outer membrane mitochondrial protein VDAC. Equally sized "Regions Of Interest” (ROIs) were analyzed with Image J, and the area was expressed in pixel units. No differences were observed between controls and IIM patients. Controls n = 3; Patients n = 4. (B). Mitochondrial complexes (I, III, IV and V) were analyzed by Western Blot in tissue samples from human skeletal muscle biopsies obtained from control and IIM patients. No differences were observed. Controls n = 3; IIM n = 3. Mean ± SEM. (TIF) [file pone.0242443.s001.tif]
