# Supplementary material for: Idiopathic inflammatory myopathy human derived cells retain their ability to increase mitochondrial function
Source: PLoS One. 2020 Nov 20;15(11):e0242443. doi: 10.1371/journal.pone.0242443 (PMC7679003; doi:10.1371/journal.pone.0242443)
Supplement: Attachment — Submitted filename: second Respond to reviewers comments 101520_al.docx [file pone.0242443.s003.docx]

**Responses to Reviewers’ Comments**

Regarding our work entitled “Idiopathic Inflammatory Myopathy Human Derivate Cells retain their ability to increase mitochondrial function” (PONE-D-20-20169) we want to thank reviewer 1 for accepting our amended manuscript and reviewer 2 for acknowledging that the quality of our work has greatly improved and that all his major concerns have been addressed.

Reviewer comments:

Reviewer #2: The authors have addressed major concerns of the manuscript and have thus improved the quality of the paper, however, a few points still need to be reviewed.

1- The authors have provided data to show that mitochondrial mass is similar between both Controls and IMM using NAO probe. Using this probe, the authors have to mitigate their interpretation of the data for a few reasons which are:

-NAO accumulates in mitochondria in a potential-dependent manner. The authors having stated that mitochondria are hyperpolarized in IMM, there's a high possibility that the dye distribution is different between CTL and IMM.

-Cardiolipin are also found in peroxisomes

I suggest the authors use other indicators of mitochondrial mass such as mtdna/nuclear dna ratio in all conditions (glucose and galactose). Indeed, several studies had shown an increase in mitochondrial turnover in galactose, I believe the paper would be greatly improved to show that the observed changes are not attributed to changes in mitochondrial mass.

***Response:*** *We thank the reviewer for this insightful comment. We agree that NAO is not the best probe to determine mitochondrial mass. Conflicted results have been published regarding its use (REF, siy no). Related to our experiments, the fact that the measurements with NAO are similar in control and IMM cells, despite IMM cells being hyperpolarized may suggest that; 1- IMM cells have way less mitochondria but given the high mitochondrial membrane potential, accumulated so much dye that the quantification is similar to control, or 2- NAO binds mainly to cardiolipin and there is no difference in mitochondrial mass.*

*As mentioned by the reviewer the best methods to quantify mitochondrial mass is by performing a mitochondrial DNA/nuclear DNA ratio. This is a technique that we want to implement in our lab but unfortunately is not working at the moment. In the current situation, we have no access to patients’ samples to culture cells and do the required experiments. All the health personnel is working on covid-19 related tasks and all other projects are on hold. We have some frozen material, but the cells were not able to grow. The only material we have available are biopsy samples. We performed Western blots of the mitochondrial complexes in biopsy samples from normal and IIM patients. In agreement with the previous analysis of VDAC performed on biopsy samples from control and IIM patients, no differences in the expression of complex I, III, IV and V were found between the samples. This data has been added as supporting figure 1. Altogether, our data suggest that no difference in mitochondrial mass is observed between normal and IIM conditions, however, we understand that better experiments can be done to show this with less doubt. We made a statement regarding this in the discussion. We add the following sentences (red) to the results and the discussion section;*

*Results*

*…”* *Nonylacridine orange (NAO) staining show no differences between control and IIM cells (fig. 2D) suggesting no differences in mitochondrial mass. To strengthen this point, we labeled biopsy samples from control and IIM patients with an antibody against the outer mitochondrial protein VDAC and we determined its expression by quantifying the number of pixels per area in confocal microscope images. As shown in the supporting figure 1A, no difference in the distribution nor in the expression were found between normal and IIM samples. In addition, using an antibody cocktail we determined the expression of the electron transport chain complexes by Western blot in biopsy samples from control and IIM patients. As shown in supporting figure 1B, no changes in the expression of complex I, III, IV and V were observed between control and IIM samples. Due to technical reasons complex II was not identified in any of our samples. These results strengthen the idea that no difference in mitochondrial mass exists between normal and IIM cells. More experiments are necessary to confirm this point.*

*Discussion*

*…”* We interpret this result as a demonstration of the IIM cell ability to adapt successfully to a metabolic stress condition. Nonylacridine orange (NAO) labeling of cells in culture, in addition to the labeling of the outer mitochondrial protein VDAC and Western blot of the electron transport chain complexes I, III, IV and V on biopsy samples from control and IIM patients, suggests that no changes in mitochondrial mass between normal and IIM samples exist. NAO binds cardiolipin, which is highly concentrated in the mitochondria independent of ΔΨm (19), however under certain circumstances it accumulates in the mitochondria in a ΔΨm-dependent fashion (20,21). To confirm that the mitochondrial mass is similar between control and IIM samples, further experiments measuring the mitochondrial/nuclear DNA ratio are necessary.

2-Authors suggest that forcing IMM cells to rely on mitochondrial synthesized ATP renders the cells more prone to death ffollowing acute Oxidative insult. The study would be strengthened by inhibition of ROS production, to further establish a direct role of ROS production in the IMM cells sensitivity to death, particularly in galactose medium culture.

***Response:*** *We agree with the reviewer. The utilization of antioxidants or other means to inhibit ROS would strengthen our conclusion. Unfortunately, we don’t have cells to perform this experiment and given the covid-19 pandemic, all the health personnel that could help us with the samples, including Dr Bozan, are now working to contain covid-19. In the discussion we have stated that experiments with antioxidants are necessary to prove the role of ROS in the cell death observed.*

*…” In the current research, we observed an increase in ROS levels in IIM-derived cells in both GLU- and GAL-media (fig. 2E and 2F). However, to be sure about the role of ROS, further experiments using molecular and/or pharmacological intervention of the redox homeostatic system are necessary.*

3-Authors have shown graph of ECAR measurement which do not show differences between Control and IMM. I believe these were done in high glucose medium. It would be interesting to show these ECAR measurements in Galactose medium.

***Response:*** *As mentioned by the reviewer, the ECAR measurements we share with the reviewers in our first respond were indeed performed in high glucose. Here we added a representative ECAR of cells growth in galactose. As shown in the figure 1 of this respond, no differences in ECAR are observed between control and IIM cells grown in galactose medium. Further experiments using the glycolysis stress kit are necessary to get more details about glycolysis in these cells. However, the lack of differences observed in these measurements suggest that no differences will be found.*

*Nevertheless, the main focus of this manuscript is on the mitochondrial function and its unexpected deleterious effect on IIM cells.*

*Figure 1. ECAR of control and IIM cells in culture.*

*We understand that we have not been able to fully respond to the observation of reviewer 2, not because we didn’t consider his/her input, but because of the catastrophe caused by covid-19, which has basically stopped all research in our country. The main substrate for our research are the samples from patients, to which we have no access now. All health personnel, including authors of this manuscript are now fighting covid-19 and non-medical personnel are not considered necessary and therefore not allowed access. The basic researchers involved in this project cannot go to the hospital. We have tried to respond to best of our ability. We also modified the discussion to reveal the weakness of our work. Nevertheless, as is, we believe our work offers new insight into a problem poorly studied and Plos One will give us the exposure we need.*
